# Effects of Cinnamaldehyde on the Cell Wall of* A. fumigatus* and Its Application in Treating Mice with Invasive Pulmonary Aspergillosis

**DOI:** 10.1155/2018/5823209

**Published:** 2018-10-04

**Authors:** Jiehua Deng, Gangsheng Wang, Jihong Li, Yile Zhao, Xiaolu Wang

**Affiliations:** ^1^Department of Dermatology, The Second Hospital of Hebei Medical University, Shijiazhuang, Hebei 050000, China; ^2^Research Department, The Second Hospital of Hebei Medical University, Shijiazhuang, Hebei 050000, China; ^3^Clinical Lab, The Second Hospital of Hebei Medical University, Shijiazhuang, Hebei 050000, China; ^4^Department of Pharmacy, The Second Hospital of Hebei Medical University, Shijiazhuang, Hebei 050000, China; ^5^The Second Hospital of Hebei Medical University, Shijiazhuang, Hebei 050000, China

## Abstract

**Background:**

The invasive pulmonary aspergillosis is a kind of high incidence of disease with difficulties in treatment, poor prognosis, and high mortality.

**Objectives:**

The study aimed to reveal the effect of cinnamaldehyde on the fungal cell wall and verify its efficacy on invasive pulmonary aspergillosis on immunosuppressed Institute of Cancer Research mice (ICR mice).

**Methods:**

ICR mice were given cyclophosphamide 200 mg.kg^−1^. d^−1^ by intraperitoneal injection for 2 days. On the 4th day, the mice were given 50* μL* of* Aspergillosis fumigatus *spore (10^7^colony form unit CFU/mL) by intranasal injection to establish immunosuppressive animal models with invasive* Aspergillosis fumigatus* infection. Then the mice in treatment group orally administered cinnamaldehyde for 14 consecutive days, while voriconazole was given to the mice in the positive control group.

**Results:**

The clearance rate of pulmonary fungi, cure rate, and reduction of 1,3-*β*-*D*-glucans in treatment group were 80.00%, 80.00%, and 81.00%, respectively while in positive control group they were 67.00%, 60.00%, and 62.00%, respectively. There were significant differences in the results between two groups as mentioned above (P<0.05). Electron microscopy showed that, in treatment group, the cell wall of* Aspergillus fumigatus* was dissolved and detached and the cell surface was incomplete. There were edema, degeneration, and necrosis in nucleus and organelle, which lead to cellular necrocytosis. The cytomembrane of* Aspergillus fumigatus* was intact, clear, and complete, whereas the cytomembrane in the positive control group disappeared. The hyphal morphology of* Aspergillus fumigatus* was deformed, but the cell wall was intact.

**Conclusion:**

Cinnamaldehyde has a good curative effect in the treatment of invasive pulmonary aspergillus infection in immunodeficient mice. It mainly affects the synthesis of 1,3-*β*-*D*-glucans from the cytoderm of* Aspergillus fumigatus* but does not affect cell wall. It would potentially be an effective and novel drug for targeted treatment of* Aspergillus fumigatus *deep infection.

## 1. Introduction

Invasive pulmonary aspergillosis infection combined with immunodeficiency is a kind of disease with difficulties in treatment, a poor prognosis, and 80-90% of mortality rate [1, 2]. The mechanism of drugs for the treatment of invasive deep fungal infection is mainly focusing on affecting the cytomembrane [3], which leads to a series of effects from side effect to toxicity and is prone to drug resistance. For example, the drug resistance rate of* aspergillus fumigatus* isolated from the clinic is up to 27% [4]. Although antifungal drugs have been used in clinical practice for many years, the mortality rate of fungal infection is still high, which has become a critical condition for inpatients and attracted great attention of medical profession. Fungal cell walls are unique to fungal cells without existing in mammalian cells and necessary for the survival of fungi. Therefore, polysaccharides of fungal cell walls, especially glucan as targeted antifungal drugs, have become a hot spot of research. Therefore, polysaccharides of fungal cell walls, especially glucan as targeted antifungal drugs, have become a hot spot of research. It is of great significance to look for the application of antideep fungal infection drugs from resource-rich traditional Chinese medicine [5–7]. Cinnamaldehyde is alternatively known as cinnamic aldehyde, phenylacrolein, and cinnamon. It is an organic compound of olefinic aldehydes extracted from the cinnamon tree. It is the main component of volatile oils and a natural trans-isomer with the form of a pale yellow oily liquid. Its chemical formula is C9H8O, and the relative molecular mass is 132.6. Many pharmacological studies in China and abroad have shown the pharmacological activities of cinnamaldehyde, such as anti-inflammatory, hypoglycemic, antitumor, and antibacterial effects, in particular, the antibacterial activity against a variety of fungi [8–11]. However, at present, the antibacterial activity of cinnamaldehyde is the result of* in vitro* experiments, while the efficacy of cinnamaldehyde in the treatment of invasive pulmonary aspergillus infection and the target mechanism of drug action are still unclear. To this end, the study adopted the methods of histology, pathology, 1, 3-format-d-glucans detection, and electron microscopy. An animal model for the treatment of invasive pulmonary aspergillosis in mice with immunodeficiency ICR was established. The efficacy and mechanism of action of cinnamaldehyde in the treatment of invasive pulmonary aspergillosis in immunodeficient mice were studied, and the preliminary results were obtained. It is expected to be an effective alternative to antifungal drugs.

## 2. Materials and Methods

### 2.1. Materials

#### 2.1.1. Strains and Animals

The Institute of Microbiology, Chinese Academy of Sciences, provided* Aspergillus fumigatus* ATCC3626. Male ICR mice aged 6–8 weeks and weighing 22–25 g were provided by Beijing Vital River Laboratory Animal Technology Co., Ltd. The quality certificate of the animal experiment was specific pathogen-free level with the license number of SCXK (Beijing) 2012-0001.

#### 2.1.2. Drugs and Reagents

Jiangxi Xuesong Natural Medicinal Oil Co., Ltd., China, provided transparent trans-cinnamaldehyde (with the purity of more than 98%). JiangSu HengRui Medicine Co., Ltd., China, provided cyclophosphamide. Ebang Pharmaceutical Co., Ltd. (Zhuhai, China), provided voriconazole. Zhanjiang A&C Biological Ltd., China, provided the (1,3)-beta-d-glucan kit. Lad Kinetics Ltd., UK, provided the dynamic test tube detector.

#### 2.1.3. Experimental Location

The experiment was performed at the Animal Experimental Center of the Second Hospital of Hebei Medical University. The animals were fed in an individual ventilated cages (IVC) laminar-flow hood with the humidity of 50%–70% at the temperature of 22°C. The Animal Care and Use Committee of Hebei Medical University approved the experiment.

### 2.2. Methods

#### 2.2.1. Model Construction and Experimental Grouping

(1) Immunosuppression group (30 mice): cyclophosphamide was intraperitoneally injected into 30 ICR mice at a dose of 200 mg.kg^−1^. d^−1^, for 2 days continuously[12].

(2) Invasive pulmonary* Aspergillus* group (30 mice): on day 4, the immunosuppressed mice were anesthetized by injecting 10% chloral hydrate (3 mL/kg) intraperitoneally, and then 50 *μ*L of* A. fumigatus ***spore** (10^7^colony form unit CFU/mL) was nasally injected. On days 14, the pulmonary tissues were used to perform microscopy and fungal culture, pathological examination, (1,3)-beta-D-glucan assay, and electron microscopic observation to establish immunosuppressive animal models with invasive* A. fumigatus* infection[13].

(3) Cinnamaldehyde treatment of invasive pulmonary aspergillosis infection group (30 mice): after 12 hours of infection with invasive pulmonary aspergillus fumigatus, oral cinnamaldehyde 240 mg.kg-1.d-1 was administered for consecutive 14 days (the effective dose for the human body × 10 times) [14]. Establish an immunosuppressed ICR mice model with invasive aspergillosis treated using cinnamaldehyde.

(4) Voriconazole-positive control group (30 mice): immunosuppressed ICR mice with Aspergillus fumigatus infection were treated with voriconazole [240 mg/(kg · d)] for 14 consecutive days. Then, lung tissue was extracted on day 14 for microscopic examination and fungal culture, histopathological examination, (1,3)-*β*-D-glucan assay, and electron microscopic observation.

(5) Saline negative control group (30 mice): on day 4, 50 *μ*L of* A. fumigatus* (1 × 10^7^CFU/mL) was nasally injected into the immunosuppressed mice. Then, the mice were orally treated with 0.5 mL of 0.9% saline after 24 h for 14 days continuously.

(6) Model group for invasive pulmonary aspergillosis infection: cinnamaldehyde treatment of invasive pulmonary aspergillosis infection group; voriconazole-positive control group and saline negative control group, taking lung tissue specimens in time for dead mice; the lung tissue samples were taken after anesthesia of the mice that did not die on the 15th day. Fungal microscopy, culture, histopathology (PAS), 1,3-*β*-D-glucans detection, and transmission electron microscopy were performed.

#### 2.2.2. Drug Preparation

2 g of Tween 80 was separately added to 97.76 ml of physiological saline, and then 240 mg of cinnamaldehyde and 240 mg of voriconazole were separately added to 2% Tween 80 physiological saline.

#### 2.2.3. Preparation of Fungal Suspension

Aspergillus fumigatus was prepared as a spore suspension of 1 × 10^7^ cfu/ml with 0.1% Tween 80 physiological saline.

#### 2.2.4. Detection of Tissue 1,3-*β*-*D*-Glucans

The content of 1,3-*β*-*D*-glucans in lung tissue was determinate by using speed turbidimetry. Totally 500 mg tissue homogenate were centrifuged at 400 g for 10 min; 100* μL* of supernatant was added to sample dilution bottle on vortex mixer for gentle mix and then placed in the tube thermostat (75°C) for 10 minutes' heating. Add 0.25 mL reagent reconstituted solution to the LPS reagent with a pipette, take 100 *μ*L of the prepared sample test solution into the reaction tube, and then add 50 *μ*L reagent solution., Then mix the solution and insert each test tube into the LKM dynamic tube tester, start the reaction, and react at 37°C for 75 minutes to read the result.

#### 2.2.5. Statistical Analysis

The statistical analysis was performed using SPSS21.0 software (SPSS, IL, USA). Continuous data were represented as mean ± standard deviation, and categorical data were represented using the chi-square test. Analysis of variance was used for comparative analysis between groups. The least significance difference *t* test was used for pair-wise comparison. The test level was set to be *α* = 0.05, and a* P* value<0.05 was considered statistically significant.

## 3. Results

### 3.1. Models of Immunosuppressed ICR Mice with Invasive* A. fumigatus* Infection

**Figure 1:**
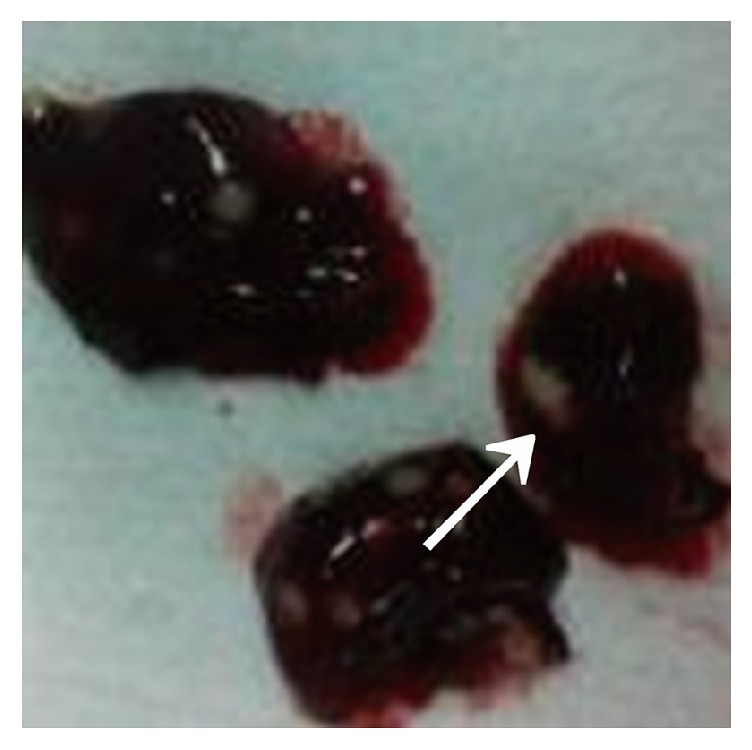
Pulmonary abscesses.

**Figure 2:**
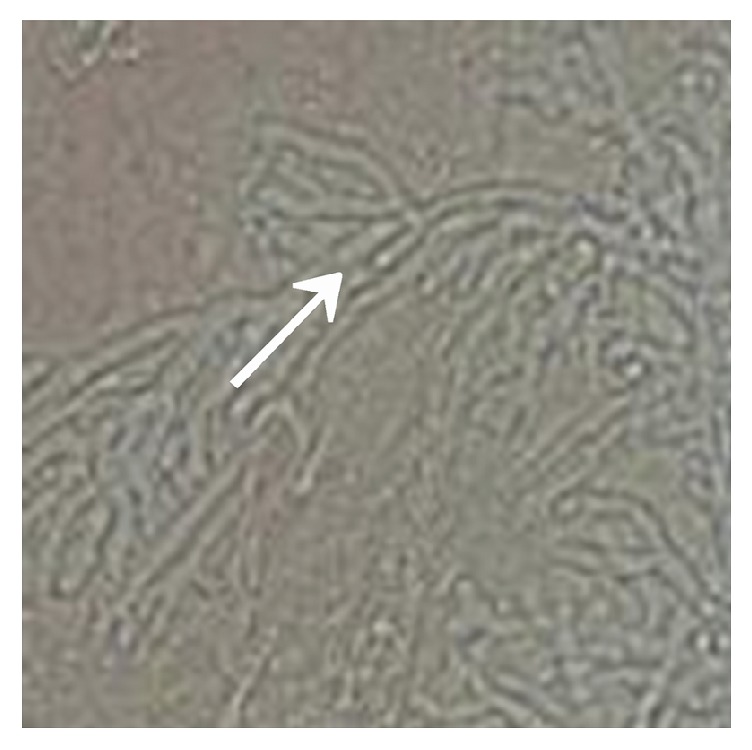
Pulmonary fungal microscopic examination (10 × 40).

**Figure 3:**
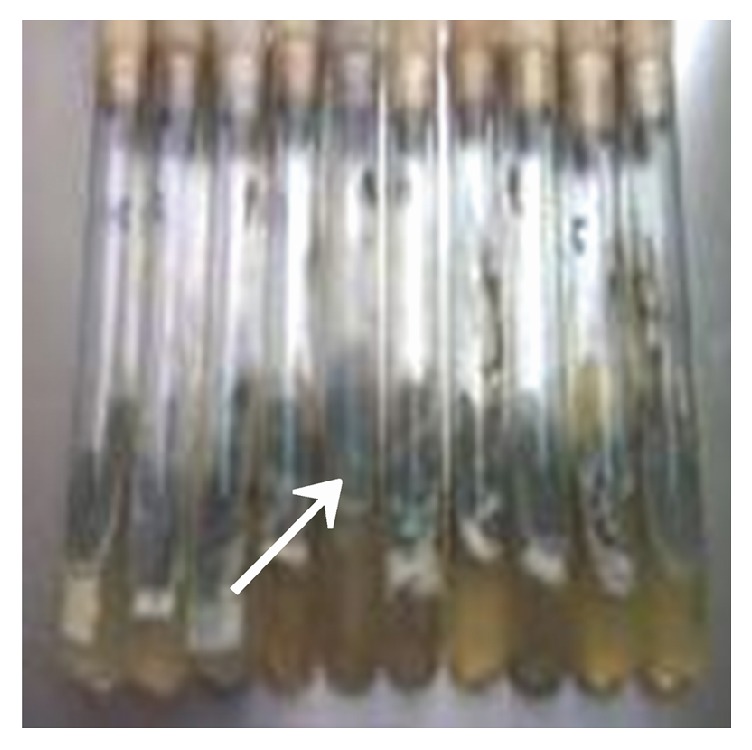
Growth of* Aspergillus fumigatus* in the lungs.

**Figure 4:**
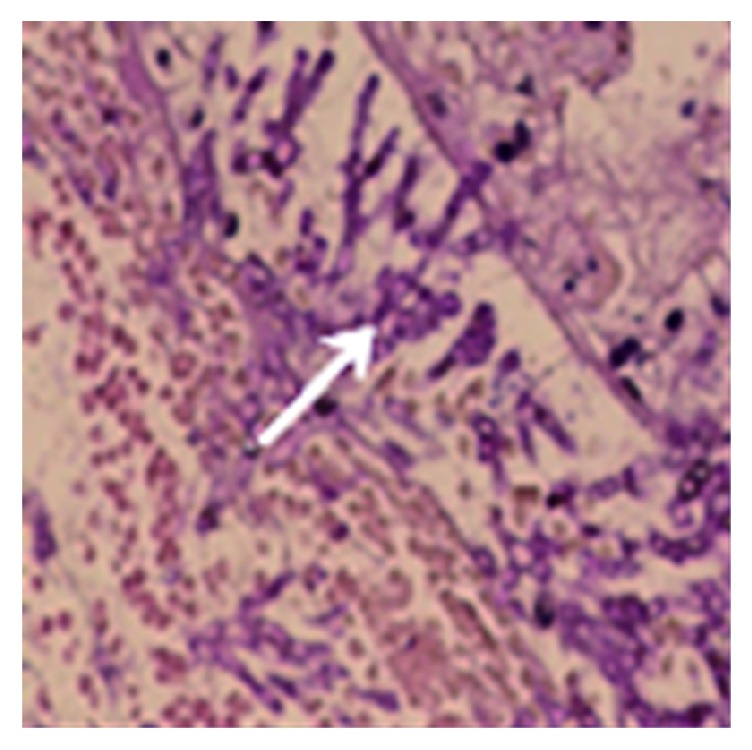
A large number of branched mycelia in the trachea.

**Figure 5:**
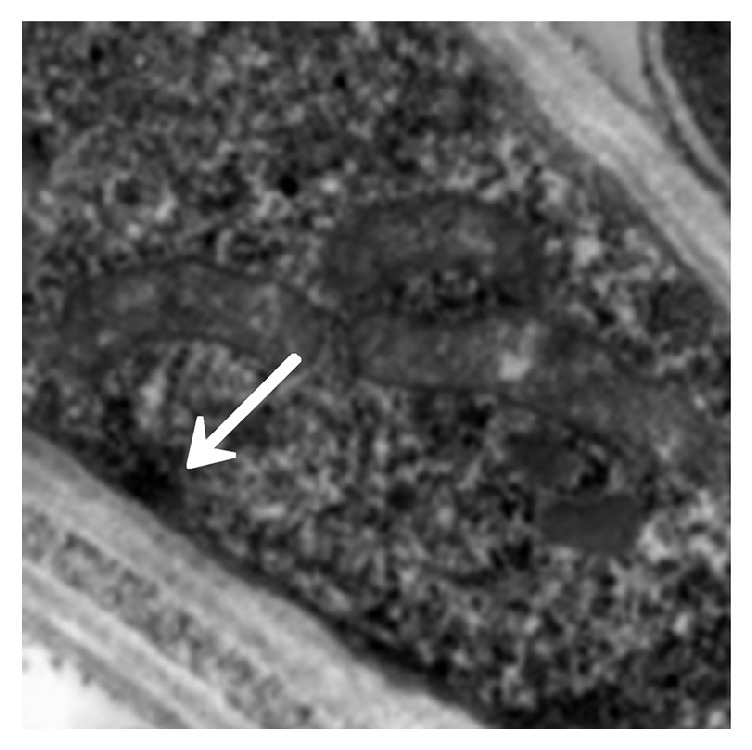
Model control (mycelium). Accelerating voltage: 80 kV; indicated magnification: 25 K×.

**Figure 6:**
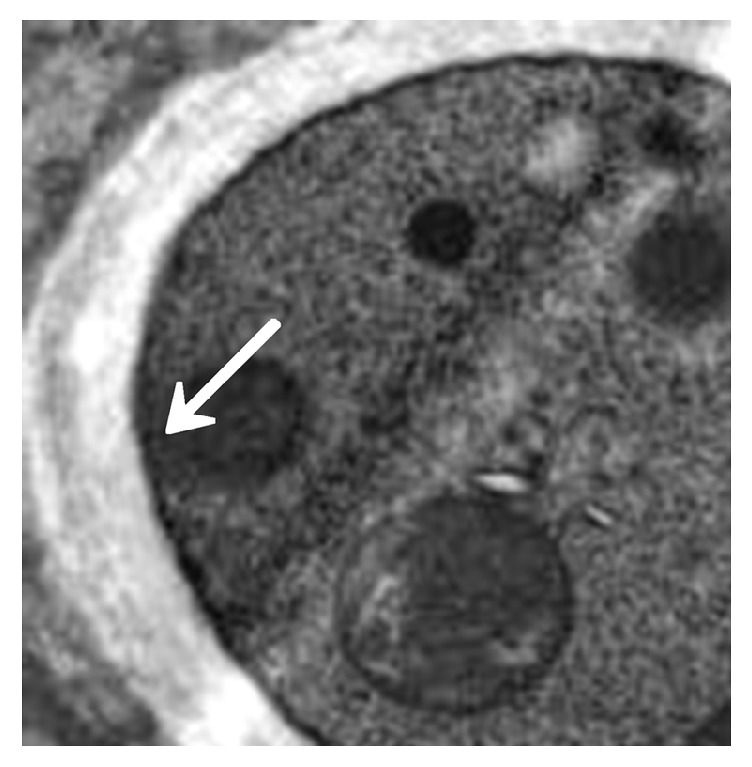
Model control (spores). Accelerating voltage: 80 kV; indicated magnification: 40K×.

The immunosuppressed mice were nasally injected 5 *μ*L of* A. fumigatus* suspension (10^7^ CFU/mL). The mice began to die after 2 days, and the mortality reached 80% after 7 days. The dead mice were anatomized, revealing multiple abscesses in the pulmonary tissues (Figure 1). Direct microscopy of the pulmonary tissues showed 45 degree branched and septate mycelium (Figure 2). Lungs cultured in Sabouraud's medium showed the growth of* A. fumigatus* (Figure 3). The pulmonary histopathological examination (PAS) under the microscope showed that a large number of mycelium and spores developed tissue necrosis and inflammatory cell infiltration (Figure 4). The pulmonary tissues were extracted from dead mice to detect (1,3)-beta-D-glucans 2–7 days after* A. fumigatus* infection. The results are shown in Table 1. Significant differences were found between the model and saline groups. The transmission electron microscopic examination of the pulmonary tissues showed that the layers of the cell wall of* A. fumigatus* hyphae were clear and complete. Furthermore, the membrane, nucleus, and contents were also clear and complete (Figures 5 and 6). The study demonstrated that models of immunosuppressed ICR mice with invasive pulmonary aspergillosis were successfully established using mycological, pathological, biochemical, and electron microscopic methods.

### 3.2. Efficacy of Cinnamaldehyde in Treating Immunosuppressed Mice with* A. fumigatus* Infection

#### 3.2.1. Mycological Efficacy

The group treated orally with cinnamaldehyde [240 mg/(kg · d)] for 14 days continuously showed that the clearance rate of pulmonary fungi was 80% after Sabouraud's culture, while it was 66.7% for the voriconazole group, suggesting that the oral administration of cinnamaldehyde was superior to the oral administration of voriconazole (Table 2). A statistically significant difference was found between the cinnamaldehyde and control groups (voriconazole, saline, model, and immunosuppression groups), indicating that cinnamaldehyde had better mycological efficacy in treating immunosuppressed ICR mice with invasive pulmonary aspergillosis.

#### 3.2.2. Pathological Efficacy

After administration of cinnamaldehyde [240 mg/(kg · d)] for 14 days continuously, the cure rate was 80%, while the cure rate of voriconazole was 66.7%. The cinnamaldehyde and voriconazole-positive control groups were statistically significantly different from the saline negative and model groups, suggesting that the cinnamaldehyde and voriconazole-positive control groups had better pathological efficacy in treating immunosuppressed ICR mice with invasive pulmonary aspergillosis. Furthermore, the cinnamaldehyde group was superior to voriconazole group (Table 3).

#### 3.2.3. Survival Curve

In the cinnamaldehyde group, cinnamaldehyde [240 mg/(kg · d)] was orally administered for 14 days continuously. The results showed that 24 immunosuppressed mice with invasive* A. fumigatus *infection survived with the survival rate of 80%. In the voriconazole group, voriconazole [240 mg/(kg · d)] was orally administered for 14 days. The results revealed that 18 immunosuppressed mice with invasive* A. fumigatus* infection survived with the survival rate of 60%. The saline group was injected 5 mL of saline, and six immunosuppressed mice with invasive* A. fumigatus* infection survived with the survival rate of 20%. After drug treatment, six mice survived in the model group with the survival rate of 20%. The cinnamaldehyde and voriconazole groups were compared with the saline and model groups, demonstrating a* P* value<0.01, while a comparison between the cinnamaldehyde and voriconazole groups revealed a* P *value <0.05. The results suggested that cinnamaldehyde significantly improved the survival time of immunosuppressed mice with invasive* A. fumigatus* infection, and it was superior to voriconazole, Figure 11.

#### 3.2.4. Detection of Tissue 1,3-*β*-*D*-Glucans

In the cinnamaldehyde group, cinnamaldehyde [240 mg/(kg · d)] was orally administered for consecutive 14 days, and the efficacy of 1,3-*β*-*D*-glucans was 81.00%, while the efficacy of voriconazole was 62.00%, suggesting that cinnamaldehyde was better than voriconazole (Table 4). Figure 11 and Table 4 revealed a comparison between the voriconazole-positive control and model groups 14 days after the oral administration of cinnamaldehyde in immunosuppressed mice with invasive* Aspergillus fumigatus* infection (*P *< 0.01), suggesting that 1,3-*β*-*D*-glucans content decreased significantly in pulmonary tissues and was lower than that in the voriconazole-positive control and model groups (2–14 days). The results demonstrated that cinnamaldehyde inhibited 1,3-*β*-*D*-glucans synthesis in pulmonary tissues, destroyed the integrity of fungal cell wall, and exerted antibacterial effects, including cell wall dissolution and cell lysis, and therapeutic effects.

#### 3.2.5. Electron Microscopic Assessment of Cinnamaldehyde in Treating Invasive* Aspergillus fumigatus* Infection

**Figure 7:**
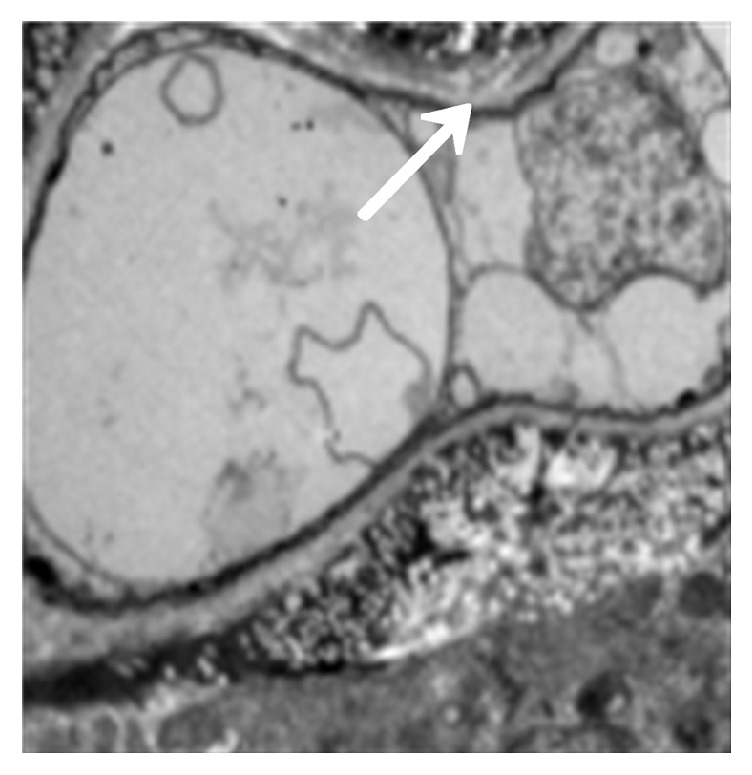
Fourteen days after cinnamaldehyde administration (mycelium). Accelerating voltage: 80 kV; indicated magnification: 8 K×.

**Figure 8:**
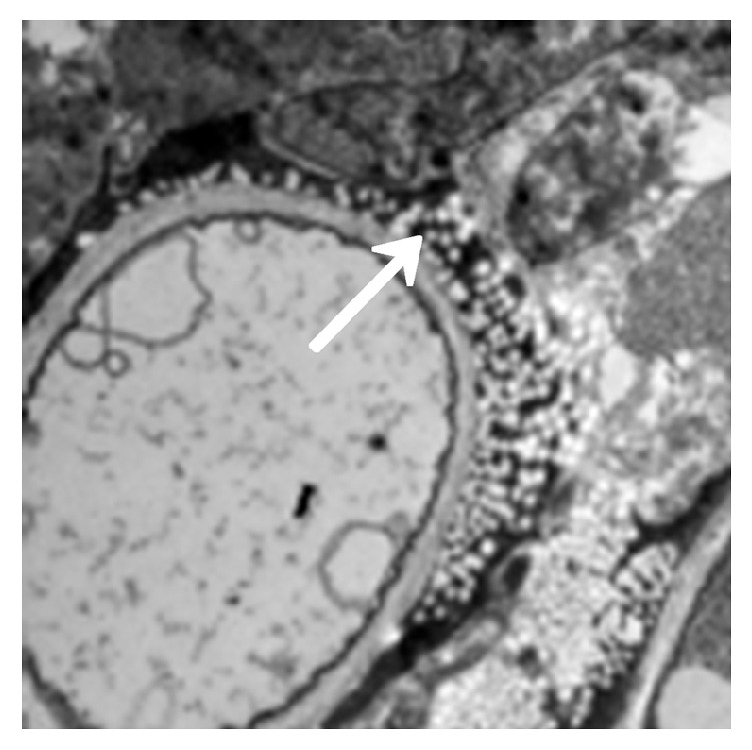
Fourteen days after cinnamaldehyde administration (spores). Accelerating voltage: 80 kV; indicated magnification: 20 K×.

**Figure 9:**
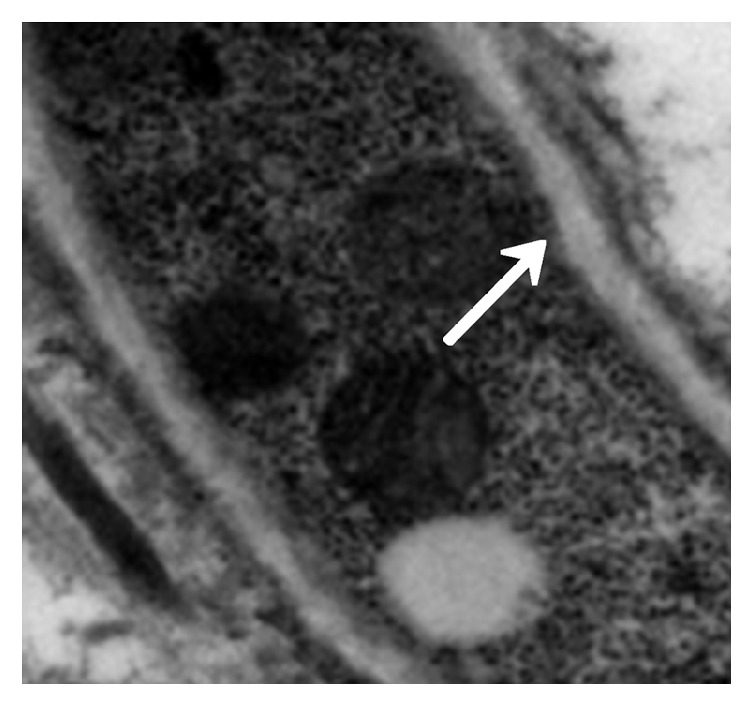
Seven days after voriconazole administration (mycelium). Accelerating voltage: 80 kV; indicated magnification: 25 K×.

**Figure 10:**
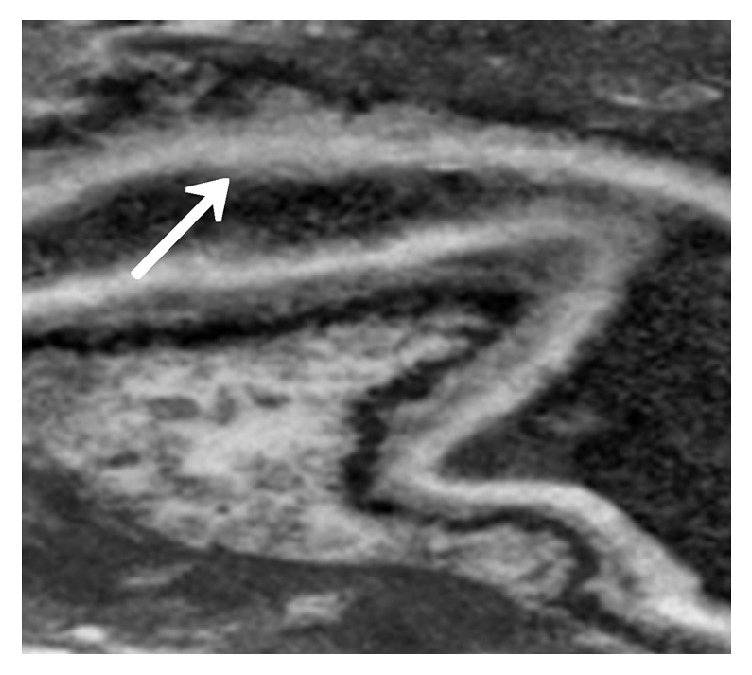
Fourteen days after voriconazole administration (mycelium). Accelerating voltage: 80 kV; indicated magnification: 25 K×.

**Figure 11:**
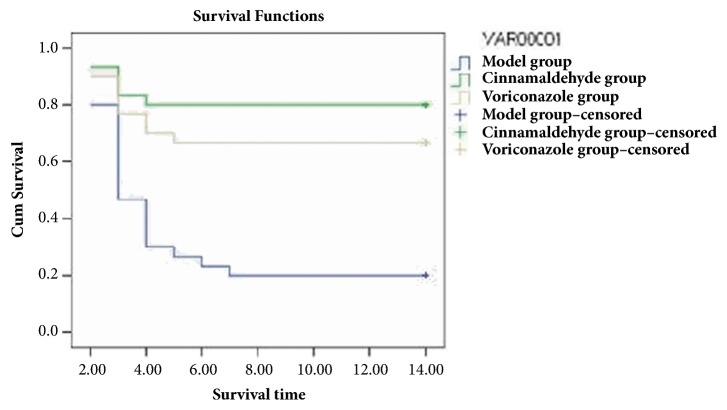
Survival functions.

The model group (30) was taken from the lung tissue specimens by transmission electron microscopy. The mycelium, spore wall, membrane, and cell contents of the tobacco mold were clear and complete (Figures 5 and 6). Oral cinnamaldehyde group (30) 240 mg.kg-1. d-1, continuous drug for 14d, and lung tissue samples were observed by transmission electron microscopy, the pathogen of Aspergillus fumigatus in the lung tissue was compared with the model comparison chart, the hyphal and spore cell walls were significantly thinner. Most of the cell walls disappeared, the surface was burr-like and smooth, and there was severe edema in the cells and around the cells. The nucleus and contents completely dissolved and disappeared, forming vacuoles, but the cell membrane remained clear and intact. (Figures 7 and 8).The results suggested that cinnamaldehyde first acted on the outer layer of the cell wall of* A. fumigatus*, interfering with the (1,3)-beta-D-glucan synthesis of the cell wall and damaging the skeletal structure and main antigenic components of the cell wall, but it did not affect the cell membrane. Subsequently, the drug directly permeated across the cell membrane to damage the nucleus and other organelles, leading to cell edema, dissolution, degeneration, and necrosis. Thus, it exerted bactericidal effect and caused cell death. However, the cell membrane was still complete, intact, and clear,

In the voriconazole group (30) 240 mg.kg-1.d-1, continuous administration for 14d, the lung tissue specimens were observed by transmission electron microscopy, the cell membrane disappeared, the hyphal morphology was deformed, but the cell wall was still intact (Figures 9 and 10). It shows that voriconazole acts on the cell membrane of Aspergillus fumigatus and has little effect on the cell wall

## 4. Discussion

Cinnamaldehyde can destroy the structural and functional integrity of fungi, because the aldehyde in its structure is a hydrophilic group, which is easily absorbed by hydrophilic groups on the surface of fungi, destroying the structure of polysaccharides and penetrating the cell wall, thus affecting the biosynthesis of cells, and inhibit growth and reproduction. Cinnamaldehyde is identified as the most active antimicrobial component and a natural antibacterial agent owing to its minimum inhibitory concentration [15]. Cinnamaldehyde can destroy the structural and functional integrity of fungi, because the aldehyde in its structure is a hydrophilic group, which is easily absorbed by hydrophilic groups on the surface of fungi, destroying the structure of polysaccharides and penetrating the cell wall, thus affecting the biosynthesis of cells. And inhibit growth and reproduction [16]. The experimental results showed that the mycological and pathological efficacy of cinnamaldehyde in the treatment of invasive pulmonary aspergillus infection in immunodeficient mice reached 80.00%, and the survival rate of mice reached 80.00% It shows that cinnamaldehyde has strong bactericidal activity against Aspergillus fumigatus infection, which can quickly remove pathogenic bacteria in body tissues, prolong the survival of severely immunodeficient animals, significantly improve the cure rate of invasive pulmonary aspergillosis infection, and reduce mortality. This experiment further confirmed that cinnamaldehyde has a strong antifungal effect not only in vitro but also has a good therapeutic effect in vivo.

1,3-*β*-*D*-Glucans mainly exists in the middle layer and outer layer of the fungal cell wall and is the main antigen component of the cell wall, accounting for 80-90% of the dry weight of the cell. It and the chitin constitute the skeleton structure of the cell wall, while maintaining cell integrity and structural stability plays an important role. Mammalian cells do not contain this ingredient, thus avoiding the toxicity of drugs to mammals [17]. Experiments show that cinnamaldehyde inhibits the synthesis of 1,3-*β*-*D*-glucans in the cell wall of Aspergillus fumigatus in lung tissue and achieves antibacterial and therapeutic effects.

The detection method of serum 1,3-*β*-D-glucans is mainly used for the diagnosis of Aspergillus and Candida infection [18, 19]. This experiment used this method to detect lung tissue specimens from invasive pulmonary aspergillosis infection in mice. The results showed that the lung tissue homogenate of the cinnamaldehyde treatment group and the model group, voriconazole group and saline group, P <0.01, cinnamaldehyde group and voriconazole group, P <0.05, and statistically significant. It is indicated that the detection method of serum 1,3-*β*-*D*-glucans is also suitable for the detection of tissue homogenate to observe the effect of the drug on the 1,3-*β*-*D*-glucans 1 of the fungal cell wall and the target position.

Cinnamaldehyde can destroy the structural and functional integrity of fungal cells, destroy the polysaccharide structure of its cell wall and penetrate the cell wall, affect cell biosynthesis, and inhibit growth and reproduction [20]. By Xie Xiaomei et al. [21], electron microscopy and isotope labeling showed that cinnamaldehyde was destroyed by the action of the cell wall of Aspergillus, and the exchange of substances inside and outside the cell was hindered, which affected the absorption of substances and the synthesis of biomacromolecules, thereby inhibiting the growth and reproduction of fungi. Wang et al. [22], by constructing a series of cell wall synthase gene knockout strains (lKS1, GHS020 and GHS003) of Saccharomyces cerevisiae, found that cinnamaldehyde can specifically inhibit the synthesis of fungal cell wall glucan and chitin. However, at present, the research on the mechanism of action of cinnamaldehyde is still in the experimental stage in vitro. In this experiment, it was found by electron microscopy that cinnamic aldehyde acts on the outer layer of the cell wall of Aspergillus fumigatus (i.e., the 1,3-*β*-*D*-glucans layer), inhibiting the synthesis of 1,3-*β*-d-glucan in the cell wall, resulting in the fact that cell wall structure is damaged, the osmotic pressure inside and outside the cell is destroyed, and the organelles are dissolved, eventually causing the cells to rupture and die. This study further confirmed that the mechanism of action of cinnamaldehyde in vivo is consistent with the mechanism of action in vitro. The target of the action is the fungal cell wall 1,3-*β*-*D*-glucans, which specifically inhibits the fungal cell wall 1,3-*β*-*D*-glucans. The synthesis achieves the purpose of inhibiting or killing fungi.

In recent years, the dramatic increase in fungal infections has threatened global health. Despite extensive research into the development of new antifungal agents, there are only a limited number of antifungal drugs on the market, conventionally used polyenes, and many azole antibiotics. Fungal agents are associated with some common side effects such as severe hepatotoxicity and nephrotoxicity. Furthermore, despite the introduction of new antifungal agents, antifungal drug resistance continues to grow and develop and complicate patient treatment and has now received attention. It has been reported [23] that cinnamaldehyde inhibits bacteria, yeast, and filamentous mold by inhibiting ATPase, cell wall biosynthesis, and changes in membrane structure and integrity. In this study, it was found by electron microscopy that cinnamic aldehyde mainly affects the biosynthesis of Aspergillus fumigatus cell wall and its structure is destroyed, but the cell membrane is still intact. The voriconazole control group shows that the targeting effect is on the cell membrane of Aspergillus fumigatus, and the cell wall is not obvious. Influence, therefore, it is easy to produce certain toxic side effects on the host. This study demonstrates that cinnamaldehyde only destroys the fungal cell wall in vivo without affecting the cell membrane and is less susceptible to toxic side effects to the host.

## 5. Summary

Cinnamaldehyde has strong anti-Aspergillus activity in vitro, has a good curative effect on invasive pulmonary aspergillosis in mice, and significantly improves the survival time of invasive pulmonary Aspergillus infection in immunodeficient mice.

Cinnamaldehyde mainly inhibits the synthesis of 1,3-*β*-*D*-glucans from the cell wall of Aspergillus fumigatus, but the cell membrane is not affected, indicating that cinnamaldehyde has a unique antifungal cell wall 1,3-*β*-*D*-glucans Glycan effect.

Cinnamaldehyde will be an ideal alternative antifungal drug for targeted treatment of deep fungal infections, but in the future research and development work on cinnamaldehyde should further focus on further study of its mechanism of action, making full use of cinnamaldehyde pharmacological activity makes it play a greater clinic.

## Figures and Tables

**Table 1:** Results of (1,3)-beta-D-glucan assay in mice that died 2–7 days after *Aspergillus fumigatus* infection (pg/mL).

Grouping	*n*	(1, 3)-beta-D-glucans	Mean value
Model group	30	4875 ± 19 to 7783 ± 45	5930 ± 36
Saline group	30	52 ± 49 to 98 ± 80	81 ± 58

The model group at 2, 4, and 6 days after administration was compared with those of the NS control group using SPSS21.0 statistical software (*P *< 0.01.

**Table 2:** Mycological efficacy of cinnamaldehyde in treating immunosuppressive mice with *A. fumigatus* infection (14 days).

Grouping	n	Microscopic examination of fungus		Fungal culture		Fungal clearance rate (%)	
		Positive	Negative	Positive	Negative	Microscopy	Culture
Cinnamaldehyde group	30	6	24	6	24	80.00	80.00
Voriconazole group	30	10	20	10	20	67.70	66.70
Saline group	30	30	0	30	0	00.00	0.00
Model group	30	30	0	30	0	00.00	0.00
Immunosuppression group	30	0	30	0	30	0.00	0.00

The fungal clearance rates of the cinnamaldehyde and voriconazole groups were compared with those of the saline, model, and immunosuppression groups using SPSS21.0 statistical software (*P *< 0.01). However, a comparison between the cinnamaldehyde and voriconazole groups suggested a *P* value <0.05.

**Table 3:** Histopathological assessment of cinnamaldehyde in treating immunosuppressed ICR mice with *Aspergillus fumigatus* pulmonary infection.

Grouping	n	Inflammatory response	Fungal-positive response	Cure rate (%)
Cinnamaldehyde group	30	6	6	80
Voriconazole group	30	10	10	66.7
Saline group	30	27	24	10
Model group	30	27	24	10

The cinnamaldehyde and voriconazole groups were compared with saline and model groups using SPSS21.0 statistical software (*P *< 0.01). However, a comparison between the cinnamaldehyde and voriconazole groups suggested a *P *value <0.05.

**Table 4:** Effect of cinnamaldehyde (pg/mL) on (1,3)-beta-D-glucans in mice with pulmonary aspergillosis infection after 2–14 days.

Grouping	*N*	(1, 3)-beta-D-glucans	*F*	*P*
Cinnamaldehyde group	30	1160.89 ± 364.96	618.441	0.000
Voriconazole group	30	3885.94 ± 845.45		
Model group	30	5930.36 ± 716.49		
Saline group	30	81.58 ± 11.89		

The cinnamaldehyde and voriconazole groups were compared with the saline and model groups using SPSS21.0 statistical software, suggesting a *P *value <0.01. A comparison between the cinnamaldehyde and voriconazole groups showed statistical significance.

## Data Availability

The data used to support the findings of this study are available from the corresponding author upon request.
